# Book Notices

**Published:** 1880-10-20

**Authors:** 


### BOOK NOTICES.

NOTES ON THE ANATOMICAL RELATIONS OF UTERINE STRUCTURES. With Surgical Remarks and Therapeutical Suggestions. By T. H. Buckler, M. D., Baltimore, Md.

A CASE OF COMPLETE INVERSION OF THE UTERUS. With Remarks Upon the Modern Treatment of Chronic Inversion. By Clifton E. Wing, M. D., Boston.

SOME PRACTICAL SUGGESTIONS IN THE TREATMENT OF DIPHTHERIA. By R. J. Nunn, M. D., Savannah, Ga., Professor Practice of Medicine in Savannah Medical College; President of Georgia Medical Society, etc.

ON PORT-WINE-MARK AND ITS OBLITERATION WITHOUT SCAR: Fourth Edition. By Balmanno Squire, London, Surgeon to the British Hospital for Diseases of the Skin. Lond, : J. A. Churchill, New Burlington Street, 1880.

NEW METHOD OF PERMANENTLY REMOVING SUPERFLUOUS HAIRS. By L. Duncan Bulkley, A. M., M. D, Physician to the Skin Department, Demilt Dispensary, New York; Attending Physician for Skin and Venereal Diseases, at the New York Hospital, Out-Patient Department, etc.

ON THE USE OF WATER IN THE TREATMENT OF DISEASES OF THE SKIN. By L. Duncan Bulkley, A. M., M. D., Physician to the Skin Department, Demilt Dispensary, New York; Attending Physician for Skin and Venereal Diseases at the Out-Patient Department of the New York Hospital; Consulting Department to the Hospital for Ruptured and Crippled, New York, etc.

ON THE NOMENCLATURE AND CLASSIFICATION OF DISEASES OF THE SKIN. With Remarks upon that Recently Adopted by the American Dermatological Association. By L. Duncan Bulkley, A. M., M. D., Physician to the Skin Department, Demilt Dispensary, New York; Attending Physician for Skin and Venereal Diseases at the Out-Patient Department of the New York Hospital.

THE AMERICAN ALMANAC AND TREASURY OF FACTS, 1880; Statistical, Financial and Political; edited by Ainsworth R. Spofford; Librarian of Congress—New York, The American News Company.

Presented to the Editors by H. H. Warner & Co., Rochester, N. Y., to whom our thanks are returned, as it contains an unusual amount of valuable information useful to everybody.

A TREATISE ON COMMON FORMS OF FUNCTIONAL NERVOUS DISEASES, by L. Putzell, M. D., Physician to the Clinic for Nervous Diseases' Randall's Island Hospital, Pathologist to the Lunatic Asylum—B, J. Cureton to Charity Hospital, etc.—New York, Wm. Wood & Co. pp. oc. 256.

There is no class of diseases more perplexing and difficult than functional nervous affections. The above book contains many valuable hints and practical suggestions which the practitioner will appreciate. We have examined the chapters on Chorea and Epilepsy with special interest: and the articles upon Neuralgia and Peripheral Paralysis are likewise very interesting and practical.

A CASE OF ACUTE PUERPERAL INVERSION OF THE UTERUS: A Clinical Description of a New Instrument Successfully Employed. With Remarks on the Mechanism of Restoration. By John Byrne, M. D., M. R. C. S. E., Surgeon-in-Chief to St. Mary's Hospital for Diseases of Women, Brooklyn; Fellow of the American Gynecological Society; of the New York Obstetrical Society; of the New York Academy of Medicine; Member of the American Medical Association, etc.; Formerly Clinical Professor of Uterine Surgery in the Long Island College Hospital; Corresponding Fellow of the Gynecological Society of Boston; Member of the Kings County Medical and Pathological Societies, etc. New York: D. Appleton & Co.

AMERICAN NEWSPAPER DIRECTORY: George P. Rowell & Co., containing acurate lists of all the newspapers and periodicals in the United States, Territories and Dominion of Canada, together with a description of the towns and cities in which they are established, New York.

Rowell's Directory has gradually grown into a large and exceedingly useful work to the business public. The book before us is a large octavo of more than 1,000 pages. The amount of labor and expense employed in getting up a work of such varied and extensive information must have been prodigious. It is probably the most complete work of the kind ever published.

A TREAITSE ON THE PRACTICE OF MEDICINE: For the use of Students and Practitioners. Robert Bartholow, M. A., M. D., L. L. D., Professor of Modern Medicine and General Therapeutics in the Jefferson Medical College of Philadelphia, etc. New York, D. Appleton & Co., 1880.

Dr. Bartholow has intended this work as a consort to his splendid contribution to Medical Literature, that of Therapentics These works are splendidly matched. The volume before us gives not only full and correct descriptions of diseases, but the treatment is enlivened under the i nstruction of of a new therapy, which must commend itself to every student. It is an honor alike to authors and the profession of the country.

